# Correlation between hematological parameters and PET/CT metabolic parameters in patients with head and neck cancer

**DOI:** 10.1186/s13014-022-02112-4

**Published:** 2022-08-13

**Authors:** Beat Bojaxhiu, Dubravko Sinovcic, Olgun Elicin, Arnoud J. Templeton, Mohamed Shelan, Jan Wartenberg, Ian Alberts, Axel Rominger, Daniel M. Aebersold, Kathrin Zaugg

**Affiliations:** 1 Department of Radiation Oncology, Inselspital, Bern University Hospital and University of Bern, Freiburgstrasse, 3010 Bern, Switzerland; 2 Department of Radiation Oncology, Stadtspital Triemli, Zurich, Switzerland; 3 Department of Nuclear Medicine, Inselspital, Bern University Hospital, University of Bern, Bern, Switzerland; 4 Department of Medical Oncology, St. Claraspital Basel and Faculty of Medicine, University of Basel, Basel, Switzerland

**Keywords:** Head and neck, Radiotherapy, PET, NLR, PLR

## Abstract

**Background:**

Systemic inflammation is predictive of the overall survival in cancer patients and is related to the density of immune cells in the tumor microenvironment of cancer, which in turn correlates with ^18^F -fluorodeoxyglucose (FDG)-positron emission tomography/computed tomography (PET/CT) metabolic parameters (MPs). The density of tumor-infiltrating lymphocytes (TILs) in the microenvironment has the potential to be a biomarker that can be used clinically to optimize patient selection in oropharyngeal head and neck squamous cell carcinoma (HNSCC). There is little to no data regarding the association of systemic inflammation with PET/CT-MPs, especially in HNSCC. This study aimed to evaluate the correlation between markers of host inflammation, namely blood neutrophil-to-lymphocyte ratio (NLR) and platelet-to-lymphocyte ratio (PLR), with the PET/CT-MPs standardized uptake value (SUV), metabolic tumor volume (MTV), and total lesion glycolysis (TLG) of the primary tumor, derived from FDG-PET/CT in patients with nonmetastatic (cM0) HNSCC before treatment. We hypothesized that NLR and PLR at baseline are positively correlated with PET/CT-MPs.

**Methods:**

A retrospective review of consecutive patients with HNSCC with a pretreatment PET/CT was performed. NLR and PLR were computed using complete blood counts measured within 10 days before the start of any treatment. The correlation between NLR and PLR with PET/CT-MPs was evaluated with Spearman's rho test.

**Results:**

Seventy-one patients were analyzed. Overall survival (OS) at 1, 2, and 3 years was 86%, 76%, and 68%. PLR was found to be correlated with MTV (rho = 0.26, *P* = .03) and TLG (rho = 0.28, *P* = .02) but not with maximum SUV or mean SUV. There was no correlation between NLR and the analyzed PET/CT-MPs. TLG was associated with worse survival in uni- and multivariable analysis, but no other PET/CT-MPs were associated with either OS or disease-specific survival (DSS). NLR and PLR were associated with OS and DSS on uni- and multivariable analysis.

**Conclusions:**

In patients with HNSCC before any treatment such as definitive radio (chemo)therapy or oncologic surgery followed by adjuvant RT, baseline PLR correlated with MTV and TLG but not with SUV. NLR was not correlated with any PET/CT-MPs analyzed in our study. Confirmatory studies are needed, and a potential interaction between tumor microenvironment, host inflammation, and FDG-PET/CT measures warrants further investigation.

**Supplementary Information:**

The online version contains supplementary material available at 10.1186/s13014-022-02112-4.

## Introduction

Radiotherapy (RT) is the standard of care for most patients with head and neck squamous cell carcinoma (HNSCC) treated definitively or adjuvantly. Besides the effects of RT through direct or indirect deoxyribonucleic acid damage, RT can also induce antitumor immune responses that contribute to indirect tumor cell killing [1, 2]. Several studies have demonstrated that tumor-infiltrating lymphocytes (TILs) are present in the tumor microenvironment in various malignant tumors [3–5]. In oropharyngeal carcinoma, TILs can identify stage I human papillomavirus (HPV)-associated patients likely to be poor candidates for treatment de-escalation [6]. In non-small-cell lung carcinoma (NSCLC), studies have shown that the tumor microenvironment correlates with ^18^F-fluorodeoxyglucose (FDG) positron-emission tomography/computed tomography (PET/CT) metabolic parameters (MPs) [7]. The therapeutic potential of radioimmunotherapy in HNSCCs is as vast as the tumor microenvironment of HNSCCs is complex [8]. This field awaits results from ongoing clinical trials providing immunological and genomic analyses from radioimmunotherapy trials, hopefully revealing information on tumor behavior and therapeutic options [8]. The expression of programmed cell death ligand-1 (PD-L1) by tumor cells and TILs have been proposed as promising prognostic biomarkers for response to immunotherapy in HNSCCs [9, 10]. In NSCLCs, Wang et al. showed an association between FDG-PET/CT-MPs and immune cell expression in the tumor microenvironment [7]. This association suggests that PET/CT-MPs could be potential predictors for selecting immunotherapy candidates for treatment with anti-programmed cell death-1/PD-L1 antibodies [7, 11]. Systemic inflammation is also correlated with cancer prognosis. Many lines of evidence indicate that markers of systemic inflammation are independent prognostic factors for survival in many malignancies [5, 12–17]. The neutrophil–lymphocyte ratio (NLR) is one such commonly used marker that has been reported as a prognostic factor [16–18]. Since the correlation between systemic inflammation and tumor microenvironment has been described [7] and owing to the scarcity of retrospective data as well as relevant literature concerning interactions between the tumor microenvironment and MPs derived from FDG-PET/CT in HNSCC, we aimed to evaluate correlations between readily available markers of host inflammation with PET/CT-MPs in patients with nonmetastatic HNSCC before definitive treatment such as surgery followed by adjuvant radio(chemo)therapy or definitive radio(chemo)therapy alone. As described in other tumor entities, e.g., breast [19] and lung cancer [7], we hypothesized that NLR and platelet-to-lymphocyte-ratio (PLR) at baseline are positively correlated with PET/CT-MPs such as standardized uptake value (SUV), metabolic tumor volume (MTV), and total lesion glycolysis (TLG) of the primary tumor.

## Methods

### Patients and complete blood count

We identified consecutive patients with HNSCC in our institutional database treated with primary or adjuvant intensity-modulated RT with or without concomitant systemic treatment between January 2007 and December 2010 at the Department of Radiation Oncology, Inselspital, Bern University. Treatment for these patients was with curative intent in all cases. Exclusion criteria were history of another malignancy within 5 years of diagnosis, prior radiation to the head and neck, non-squamous cell carcinoma histology, distant metastases, lack of differentiated complete blood count within 10 days before oncologic surgery or starting RT, and lack of PET/CT before beginning of any treatment. Pretreatment complete blood counts with differential values were used to calculate NLR and PLR. NLR was calculated by dividing absolute neutrophil count by absolute lymphocyte count; PLR was calculated by dividing absolute thrombocyte count by absolute lymphocyte count. Patients with leukocytosis caused by other reasons (e.g., infection, steroid use, etc.) were excluded from the analysis. This study was approved by the local ethics committee (289/2014) and was conducted in full accordance with ethical principles, including the World Medical Association Declaration of Helsinki (version 2002) and local legislation.

### PET/CT acquisition

All patients fasted for at least 6 h before intravenous administration of FDG. All examinations were performed on one of two cross-calibrated BIOGRAPH-mCT PET/CT scanners (Siemens, Erlangen, Germany). Image acquisition started 60 min after tracer injection, and patients were studied as follows. First, a non-contrast-enhanced CT scan was performed from the skull base to mid-thigh with arms elevated using the following parameters: slice thickness of 5 mm; increment of 3.0 mm; soft tissue reconstruction kernel; maximum of 100 keV and 90 mAs by applying CARE kV and CARE Dose. Immediately after the CT scan, a whole-body PET (pelvis to vertex) was acquired in three dimensions (3D; matrix 200 × 200) with a zoom factor of 1. For each bed position (16.2 cm, overlapping scale 4.2 cm), a 2-min acquisition time with a 15.5 cm field of view was used. The emission data were corrected for randoms, scatter, and decay. Reconstruction was conducted with an ordered subset expectation maximization algorithm with four iterations/21 subsets and Gauss filtered to a transaxial resolution of 5 mm at full width at half maximum. Attenuation correction was performed using the low-dose non-enhanced CT data. After the whole-body PET/CT imaging, a dedicated head and neck acquisition was performed from the cranial vertex to the thoracic inlet in an arm-down position for use in the SUV and MTV calculations. PET images were obtained in two table positions (10 min per table position). The PET data were reconstructed iteratively (four iterations/21 subsets) and an image matrix of 512 × 512 pixels was used. The head and neck CT scan parameters were: 120 kV, 80 mA, helical thickness of 3 mm, and field of view of 78 cm.

### PET/CT analysis

FDG-PET/CT data were analyzed using an appropriate workstation and software (SyngoVia; Siemens, Erlangen, Germany). PET/CT images were visually interpreted for increased FDG uptake by the first author (BB) and a board-certified nuclear medicine physician with more than 15 years (JW) of clinical experience in oncological PET/CT. Compared to the FDG uptake of background and liver, the primary tumor with focally increased FDG uptake was considered positive for carcinoma. Semiquantitative analysis was based on manually placed regions of interest around the primary tumor with increased FDG uptake to calculate the MTV. The mean and maximum SUV values (SUVmean and SUVmax, respectively) of the lesions were calculated according to the formula:

SUV = tissue concentration (Bq/mL)/(injected dose (Bq)/body weight (kg)).

The region of interest was delineated on the primary tumor site. SUVmax, SUVmean, and MTV were measured from 3D isocontour at 40% of maximal pixel value [20]. TLG was calculated by multiplying SUVmean by MTV for the lesion [21].

### Treatment and follow-up

As previously published, the standard treatment was based on institutional policies following the multidisciplinary tumor board decision [22, 23]. The standard treatment for oral cavity cancer was to perform surgery followed by adjuvant RT [22, 24, 25]. In oropharyngeal, hypopharyngeal, and laryngeal cancers, the joint recommendation of the multidisciplinary meeting was primary RT [26]. The standard concomitant therapy consisted of cisplatin 100 mg/m^2^ on day 1 in 3-week intervals for all patients. Patients not deemed medically fit for cisplatin chemotherapy because of pre-existing comorbidities were evaluated for weekly treatment with the monoclonal antibody cetuximab [27] or carboplatin every 3 weeks. In a few cases of induction chemotherapy, the triplet of cisplatin, docetaxel, and 5-fluorouracil was used. Patients were regularly followed, and toxicities were graded according to the National Cancer Institute Common Terminology Criteria for Adverse Events (version 4.03) [28].

### Statistical analysis

NLR was calculated by dividing absolute neutrophil count by absolute lymphocyte count measured in peripheral blood. PLR was calculated by dividing absolute thrombocyte count by absolute lymphocyte count. Thresholds were set as described by De Felice et al. [29]. Frequencies and percentages were reported for categorical variables; continuous variables were reported as medians with range or interquartile range. The endpoints of the study were the correlation between NLR and PLR with PET/CT-MPs, overall survival (OS), and disease-specific survival (DSS). The correlation between NLR and PLR with PET/CT-MPs (i.e., SUVmax, SUVmean, MTV log, TLG log) was examined using Spearman's rho test. Time to event was calculated for OS and DSS from the start of RT to death, with censoring patients without such events at the last follow-up. The median time to event was estimated using the Kaplan–Meier method. The prognostic value of NLR and PLR and other variables (i.e., age, sex, tumor localization, SUVmax, MTV, TLG) was assessed by univariable Cox regression-analysis. Subsequently, a multivariable analysis with backward elimination was performed, including all variables with a *P*-value < 0.05 in the univariable analysis. Analyses were carried out using JMP^®^ version 14.3.0 (SAS Institute Inc., Cary, NC, USA). The threshold for statistical significance was set at *P* < 0.05, and no correction for multiple testing was performed.

## Results

A total of 189 patients were identified, of whom 118 patients (62%) were excluded. Of these, 105 were excluded because no pretreatment PET/CT was available. In 12 patients, the PET/CT scan was obtained after surgery. The remaining patient received induction chemotherapy before his PET/CT scan. Thus, a total of 71 patients with a pathologically proven diagnosis of oral cavity, oropharyngeal, hypopharyngeal, or laryngeal cancer were eligible and included for analysis. Eight patients underwent postoperative treatment, 6 of them being OCC patients having the standard of care treatment with primary surgery and adjuvant RT. The other 2 patients received a postoperative RT after an oncologic tonsillectomy could be performed. In 4 patients, due to the locally advanced stage and the inoperability of the primary tumor, it was not possible to distinguish between an oropharyngeal and an hypopharyngeal origin. Therefore we defined these primary tumors as multicompartmental. The median follow-up was 41 months (range 6–71 months). Patient and disease characteristics are presented in Table 1. The majority of patients were male (86%) and had good performance status (Karnofsky performance status > 70). Median NLR and PLR were 3.5 (IQR 2.1–4.7) and 195 (IQR 133–249), respectively.

**Table 1:** Patient and disease characteristics

Age	
Median (range), years	61 (46–83)
*Gender, n (%)*	
Female	10 (14)
Male	61 (86)
*Smoking status*	
Never smoker	5 (11)
Previous smoker	12 (26)
Current smoker	29 (63)
Missing data	25
*Karnofsky performance status*	
Median (range)	90 (60–100)
> 70, *n* (%)	60 (85)
≤ 70, *n* (%)	11 (15)
*Oncological resection of primary tumor*	
Yes	8 (11)
No	63 (89)
*Induction chemotherapy*	
Yes	7 (10)
No	64 (90)
*Concomitant systemic therapy*	
None	9 (13)
Cisplatin or carboplatin	50 (77)
Cetuximab	12 (17)
*Site of primary tumor, n (%)*	
Oral cavity	6 (8)
Oropharynx	28 (39)
Hypopharynx	19 (27)
Larynx	14 (20)
Multicompartmental	4 (6)
*UICC stage, n (%)*	
II	2 (3)
III	11 (16)
IV	58 (81)
*Tumor grade, n (%)*	
G1	1 (1)
G2	38 (54)
G3	32 (45)
*Metabolic tumor volume, MTV (cc)*	
Median (range)	6.5 (1.7–43)
*Standard uptake value, SUVmax*	
Median (range)	15.7 (4.2–34.3)
*Tumor lesion glycolysis, TLG*	
Median (range)	54 (8–471)
*Neutrophil-to-lymphocyte ratio, NLR*	
Median (range)	3.47 (0.8–19.8)
*Platelet-to-lymphocyte ratio, PLR*	
Median (range)	195 (59–951)

*UICC* Union for International Cancer Control

### Correlation between NLR and PLR with MPs

**Fig. 1:**
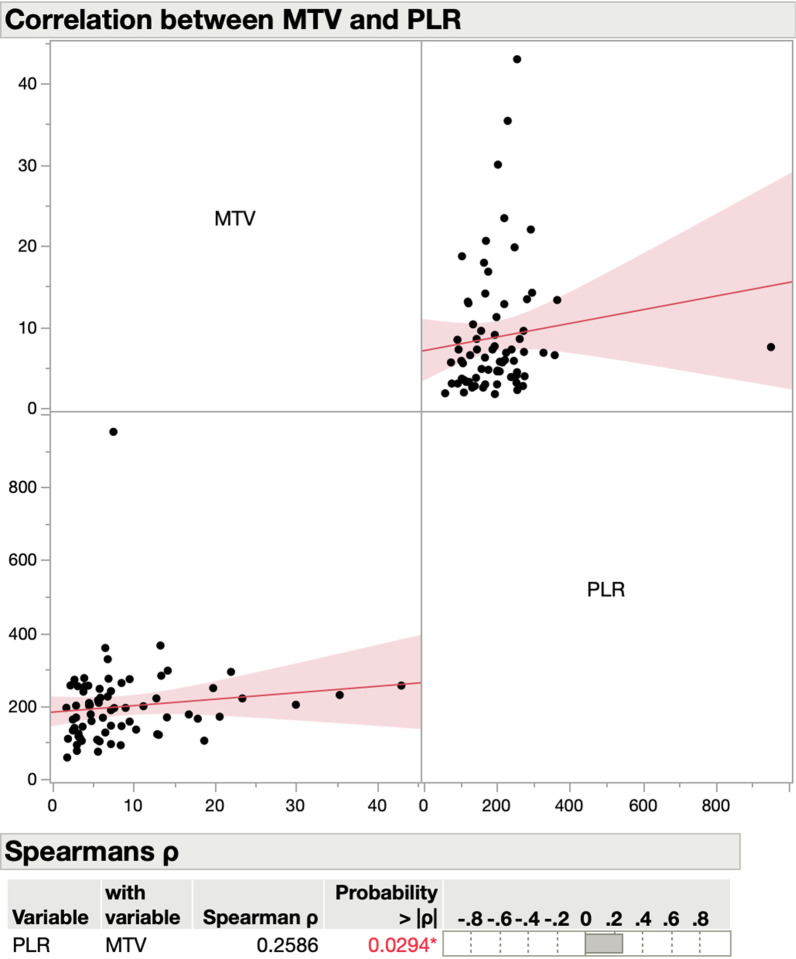
Scatter diagram for correlation between PET and hematological parameters. MTV = metabolic tumor volume; PLR = platelet-to-lymphocyte ratio

**Fig. 2:**
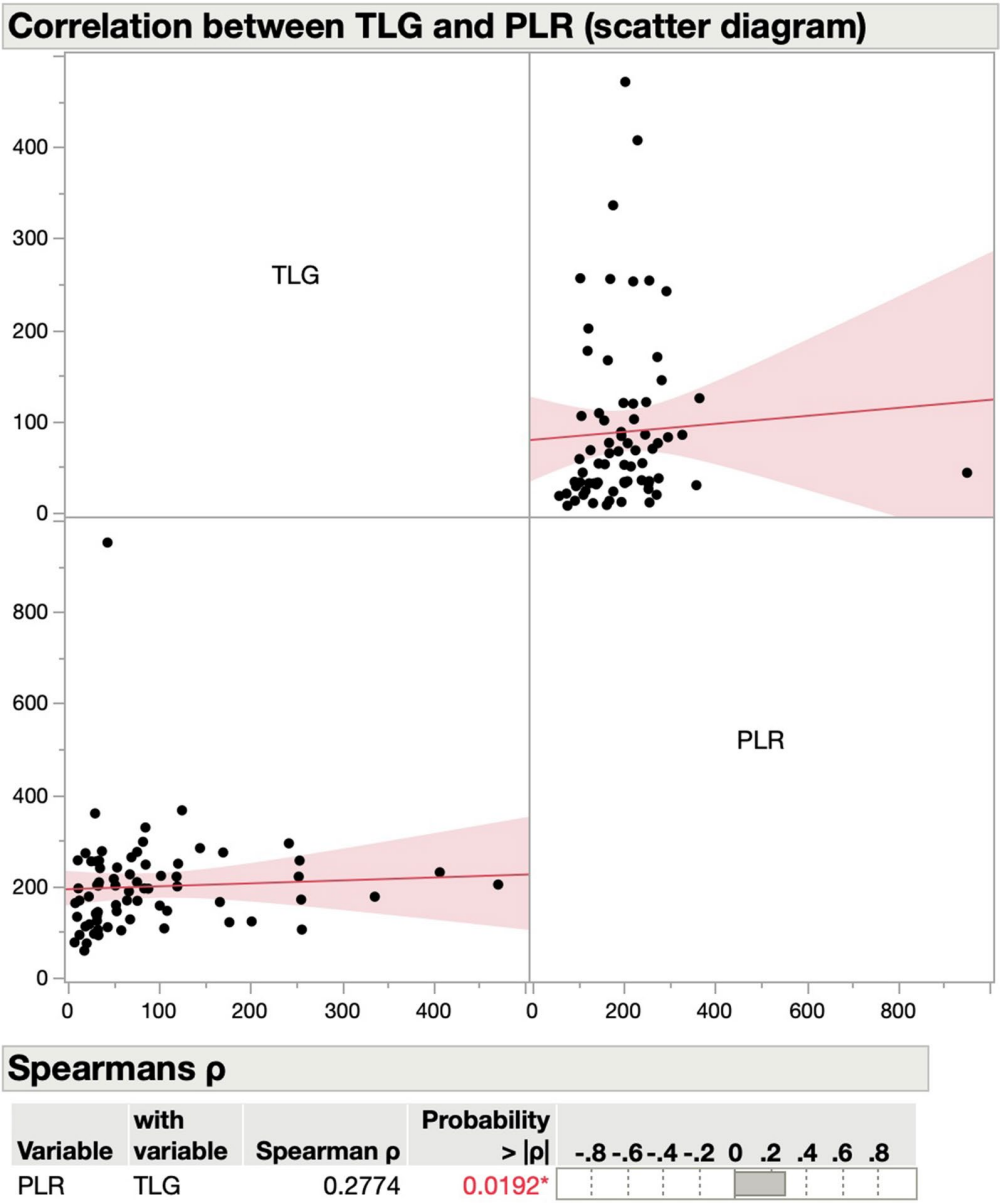
Scatter diagram for correlation between PET and hematological parameters. TLG = total lesion glycolysis; PLR = platelet-to-lymphocyte ratio

Median MTV, SUVmax, and TLG were 6.5 (IQR 3.7–11.2), 15.7 (IQR 10.2–18.6), and 54 (IQR 31.6–108.7), respectively (Table 1). NLR did not correlate with any of the PET/CT-MPs, while there was a statistically significant correlation between PLR and MTV (rho 0.26, *P* = 0.03) (Fig. 1), and between PLR and TLG (rho 0.28, *P* = 0.02) (Fig. 2) but not with SUVmax or SUVmean (Table 2). We repeated the analysis, excluding oral cavity carcinomas. The results examining the correlation of NLR and SUVmax remained unchanged (Additional file 1: Figure S1). For a solid calculation of the correlation of NLR and SUVmax for oral cavity carcinomas alone, the sample size (N = 6) is not large enough (Additional file 1: Figure S2).

**Table 2:** Correlation between PET and hematological parameters

	SUVmax	SUVmean	MTV	TLG
NLR	Rho = 0.01, P = 0.92	Rho = 0.01, P = 0.96	Rho = 0.21, P = 0.08	Rho = 0.17, P = 0.16
PLR	Rho = 0.05, P = 0.69	Rho = 0.07, P = 0.54	Rho = 0.26, P = 0.03*	Rho = 0.28, P = 0.02*

*NLR* Neutrophil-to-lymphocyte ratio, *PLR* platelet-to-lymphocyte ratio, *MTV* metabolic tumor volume, * = statistically significant, *P* value

### Predictors of DSS and OS

**Fig. 3:**
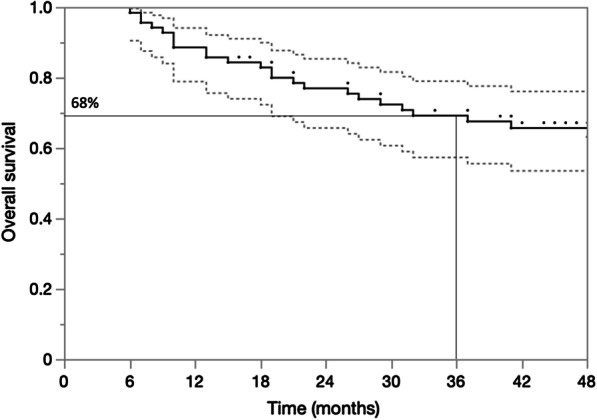
Kaplan–Meier plot for overall survival at 3 years

**Fig. 4:**
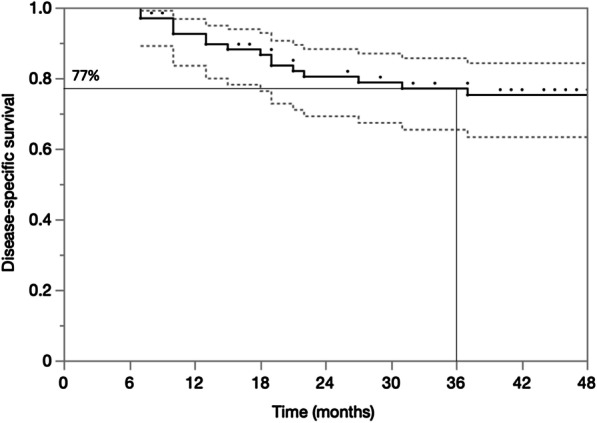
Kaplan–Meier plot for disease-specific survival at 3 years

At 1, 2, and 3 years, the OS was 86%, 76%, and 68% (Fig. 3). The DSS at 1, 2, and 3 years was 90%, 81%, and 77%, respectively (Fig. 4). On univariable analysis, TLG > 32 was associated with worse survival (*P* = 0.003). No other PET/CT-MPs were associated with either OS or with DSS. In contrast, NLR and PLR were associated with OS and DSS (Table 3). NLR, PLR, and TLG remained statistically associated with OS on multivariable analysis (Table 3).

**Table 3:** Univariable and multivariable analysis

Variable	OS		DSS	
	HR (95% CI)	*P* value	HR (95% CI)	*P* value
*Univariable*				
*Age, years*				
≤ 60	0.73 (0.32–1.66)	0.46	1.04 (0.39–2.80)	0.93
> 60– ≤ 70	1.10 (0.49–2.44)	0.82	0.97 (0.35–2.66)	0.95
> 70	1.37 (0.55–3.43)	0.5	0.99 (0.28–3.46)	0.98
*Gender*				
Male	0.79 (0.27–2.32)	0.67	0.73 (0.21–2.56)	0.63
*Tumor localization, yes (vs no)*				
Oral cavity	2.34 (0.65–7.69)	0.2	3.11 (0.87–11.13)	0.08
Oropharynx	0.59 (0.25–1.42)	0.24	0.68 (0.24–1.96)	0.47
Hypopharynx	0.93 (0.38–2.24)	0.86	0.89 (0.27–2.76)	0.84
Larynx	0.92 (0.35–2.46)	0.87	0.52 (0.12–2.78)	0.38
Multicompartmental	3.56 (1.05–12.02)	0.004*	3.47 (0.79–15.33)	0.1
T and N classification, yes (vs no)				
c/pT3–4	0.86 (0.38–1.95)	0.72	0.65 (0.24–1.74)	0.39
c/pN2b–3	1.12 (0.47–2.67)	0.81	1.92 (0.55–6.74)	0.31
AJCC stage IV	1.85 (0.55–6.18)	0.32	1.83 (0.42–8.10)	0.42
Concomitant systemic therapy, yes (vs no)				
Any	0.48 (0.16–1.42)	0.22	0.32 (0.10–1.01)	0.08
Platin–based	0.62 (0.27–1.41)	0.26	0.62 (0.22–1.71)	0.37
Cetuximab	1.19 (0.44–3.17)	0.74	0.68 (0.16–3.01)	0.60
*NLR (IQR, 2.1–4.7)*				
> Median (= 3.5)	2.27 (1.00–5.14)	0.05	2.12 (0.77–5.85)	0.15
> 2.1	1.41 (0.53–3.77)	0.49	0.76 (0.26–2.18)	0.61
> 4.7	3.44 (1.55–7.62)	0.002*	3.30 (1.22–8.93)	0.018*
*PLR (IQR, 133–249)*				
> Median (= 195)	2.63 (1.13–6.12)	0.0246*	2.71 (0.94–7.82)	0.06
> 133	5.12 (1.20–21.7)	0.027*	6.53 (0.86–49.5)	0.07
> 249	2.48 (1.09–5.66)	0.0307*	3.25 (1.20–8.76)	0.0201*
*SUVmax (IQR, 10.2–18.6)*				
> Median (= 15.7)	0.92 (0.42–2.02)	0.84	1.32 (0.49–3.53)	0.59
> 10.2	1.84 (0.63–4.5.38)	0.26	1.54 (0.44–5.40)	0.50
> 18.6	1.32 (0.55–3.18)	0.53	1.09 (0.35–3.37)	0.89
*MTV (IQR, 3.7–11.2)*				
> Median (= 6.5)	1.98 (0.89–4.12)	0.1	2.22 (0.80–6.11)	0.12
> 3.7	2.55 (0.76–8.57)	0.13	2.61 (0.59–11.5)	0.21
> 11.2	1.31 (0.55–3.14)	0.55	1.54 (0.54–4.44)	0.42
*TLG (IQR, 32–109)*				
> Median (= 54)	1.90 (0.54–2.61)	0.67	1.47 (0.55–3.96)	0.44
> 32	4.97 (1.17–21.1)	0.029*	6.76 (0.89–51.2)	0.06
> 109	1.76 (0.76–4.10)	0.19	1.71 (0.59–4.91)	0.32
Multivariable				
*Model NLR*				
Multicompartmental	5.05 (1.43–17.8)	0.0118*	–	–
NLR > 4.7	3.94 (1.73–8.97)	0.0011*	–	–
*Model PLR*				
Multicompartmental	7.10 (1.82–27.7)	0.0048*	–	–
PLR > median (= 195)	3.59 (1.40–9.20)	0.0078*	–	–
*Model TLG*				
Multicompartmental	3.93 (1.16–13.3)	0.0279*	–	–
TLG > 32	5.18 (1.22–22.0)	0.026*	–	–

*NLR* Neutrophil-to-lymphocyte ratio, *PLR* platelet-to-lymphocyte ratio, *MTV* metabolic tumor volume, * = statistically significant

## Discussion

In this retrospective analysis of 71 patients with HNSCC with a relatively long follow-up of 41 months treated with definitive or adjuvant radio(chemo)therapy after oncologic surgery, we found a statistically significant correlation between pretreatment PET/CT-MPs and hematological parameters (HPs; i.e., NLR and PLR). To our knowledge, the present study results are the first to show in patients with HNSCC that MTV and TLG correlate with pretreatment systemic inflammatory parameters, i.e., PLR. These findings align with the few other studies analyzing relationships between PET/CT-MPs and HPs in different tumor entities (Table 4). A positive correlation between HPs and PET/CT-MPs has previously been demonstrated in carcinomas of the rectum [21], esophagus [20], cervix [30], breast [19], and lung [7, 31]. In colorectal cancers, NLR was correlated with SUV, MTV, and TLG [21], except in the recurrent setting [32]. In esophageal carcinomas, NLR correlated with MTV but not with SUV [20]. In cervical carcinomas, a correlation was described between NLR and MTV with PLR and TLG [30]. In lymph node-positive breast carcinomas, a correlation between NLR and SUV and TLG of regional metastases in the axilla was shown [19]. Similarly, correlations between NLR, PLR, SUV, and MTV could also be demonstrated in small cell [31] and non-small cell [7] lung carcinomas.

**Table 4:** Selection of studies reporting on PET/CT-MPs and HP

Year	Author	N	Histology	PET/CT-MP	HP	Association/ Correlation	Purpose	Results
2019	Xu et al	231	Colorectal cancer	MTV, TLG, SUV	NLR, PLR, PLT, LMR	NLR and LMR with SUV, MTV, TLG	Correlation of pre-treatment HPs with PET/CT-MP and estimate the prognostic value of both.	NLR and LMR correlated with SUVmax, MTV, and TLG. NLR in M1 higher than M0. LMR in M1 lower than M0. OS benefit with low MTV, low NLR and high LMR.
2019	Abu-Shawer et al	264	Endometrial, ovarian cervical cancer,	Na	ANC, AMC, MCR PLR, NLR	Na	Association of NLR, MLR, PLR, and OS in advanced gynecological cancers	High baseline NLR (≥ 4.1) and high baseline PLR (≥ 0.3) with more distant metastases
2019	Can et al	129	Invasive ductal carcinoma	MTV, TLG, SUV	MPR, NLR, ER, PR, Her2, Ki67	NLR with SUV and TLG None for ICH	Relation of MTV of primary mass and ALN to molecular subtype, IHC, and inflammatory markers	ALN was associated with MTV and TLG. NLR correlated with primary mass and ALN PET parameters. PET parameters did not change with respect to molecular subtype or IHC markers. Primary mass and ALN metastasis PET parameters showed significant positive correlations for TLG and SUVmax
2019	Mirili et al	54	Small cell lung cancer	MTV, TLG, SUV	NLR	NLR with SUV, TLG, MTV	Evaluation of NLR with PET–CT MPs	NLR ≥ 4, MTV ≥ 60, had lower OS and PFS. Correlation between NLR and SUV, TLG, and MTV
2019	Du et al	89	Cervical cancer	MTV, TLG, SUV	NLR, PLR, HS	NLR and PLR with MTV, TLG, and SCC-ag	Investigation of optimal PET parameters and percentage of SUVmax (%SUVmax) thresholds for tumor recurrence evaluation, and the relationship with hematological parameters in patients with LACC	MTV and TLG had slight-to-moderate correlations with SCC-ag, NLR and PLR
2018	McSorley et al	33	Preoperative colorectal cancer	MTV, TLG, SUV	NLR, mGPS	None	Relationship between PET-CT MPs and host systemic inflammation	There was no association between 18 F-FDG-PET/CT measures of tumor metabolism and systemic inflammation in the 33 preoperative patients
2018	McSorley et al	70	Colorectal cancer (reurrence)	MTV, TLG, SUV	NLR, mGPS	NLR and mGPS with SUV, MTV, TLG	Relationship between PET-CT MPs and host systemic inflammation	Patients with NLR ≥ 5 had a higher SUV, MTV, and TLG. Patients with mGPS of 1–2 had a higher SUV, MTV, and TLG
2015	Sürücü et al	53	Esophageal cancer	MTV, SUV	NLR, PLR, MPN	NLR with MTV. None for SUV	Correlation NLR, PLR, and MPV, with SUVmax, and MTV	NLR was correlated with MTV, SUVmax was not correlated with hematological parameters
2019	Wang et al	122	Non-small cell lung cancer	MTV, SUV	ICH	SUV with IHC, NLR, PLR	Correlation between PET/CT-MP and intra-tumor immunomarkers' expression	SUV values have significant variations for different EGFR statuses (wild vs mutant type), NLR, and PLR. Correlation between SUV and CD8 tumor-infiltrating lymphocytes, CD163 tumor-associated macrophages, and Foxp3-regulatory T cells (Tregs), as well as PD-1 and PD-L1
2021	Present study	71	Head and neck squamous cell carcinoma	SUV, MTV, TLG	NLR, PLR	PLR with MTV and TLG	Correlation between NLR and PLR with SUVmax, SUVmean, MTV, and TLG	PLR correlates with MTV and TLG. SUVmax and SUVmean do not correlate with hematological parameters

Patient number (N), positron emission tomography/computed tomography (PET/CT), metabolic parameter (MP), hematological parameter (HP), standardized uptake value (SUVmax), metabolic tumor volume (MTV), tumor lesion glycolysis (TLG), lymphocyte-to-monocyte ratio (LMR), monocyte-lymphocyte ratio (MLR), immunohistochemistry (ICH), axillary lymph node (ALN), progression-free survival (PFS), overall survival (OS), recurrence-free survival (RFS), squamous cell carcinoma antigen (SCC-ag), ﻿modified Glasgow Prognostic Score (mGPS), epidermal growth factor receptor (EGFR), tumor-associated macrophages (TAMs), mean platelet volume (MPV), not applicable (na)

Overall, the limited number of studies published on this topic have mainly described a correlation between NLR and SUV and MTV, and less frequently between PLR and SUV. Interestingly, we found no correlation between NLR and SUV in our analyses. In contrast, the analysis of PLR with MTV and TLG shows a significant positive correlation, albeit not strong. The same results are provided by Sürücü et al., showing no correlation between NLR and SUV [20] in esophageal cancer. The investigators of this work attribute this to the relatively small cohort (n = 52) they analyzed. How the interaction of the tumor microenvironment and FDG-PET/CT could influence treatment decisions has also been evaluated. In NSCLCs, Wang et al. described a correlation between PD-L1-expression and PET/CT-MPs. This correlation between SUVmax and immune cell expression in the tumor microenvironment of NSCLC suggests that SUVmax on FDG-PET/CT could be predicting for immunotherapy receiving patient selection [7]. Several studies have demonstrated that TILs are present in the tumor microenvironment in various malignant tumors [3–5]. In oropharyngeal carcinoma, TILs can identify stage I HPV-associated patients likely to be poor candidates for treatment de-escalation [6]. An interplay between systemic inflammatory parameters and tumor microenvironment has also been shown. In gastric cancer, systemic inflammation is associated with the density of CD4 + lymphocytes in the tumor microenvironment [33]. We investigated whether systemic inflammatory parameters correlate with PET/CT MPs to potentially provide indirect information about the tumor microenvironment with easily accessible blood tests in combination with PET/CT-MPs. Hitherto, such analyses have not been performed in HNSCC using FDG-PET/CT. We propose that such findings would be particularly prescient in oligometastatic disease. For example, it might be possible to identify lesions that respond less to immunotherapy. FDG-PET/CT, in combination with NLR and PLR, might identify candidate lesions amenable to stereotactic radiation. We note that histopathological analysis is not always available for several patients, either for local lesions, which are not easily biopsied due to their anatomical location, or for distant metastases.

Furthermore, primary tumor and nodal metastases show poor to fair agreement when comparing biopsy material and resection tissue [34]. Predictive biomarkers, such as the composite positive score developed by Roach et al. in 2017 [35], have the potential to surmount some of these challenges and have shown promise in NSCLC [34, 36, 37]. The idea of using HPs in combination with PET/CT-MPs in patients with oligometastatic disease as a clinically useful prognostic tool for therapy response of a metastasis that is difficult to access through biopsy holds potential, not only in NSCLC but also in HNSCC.

More is known about the relationship between systemic inflammatory parameters and outcomes. In multiple studies, retrospective analyses and meta-analyses have shown that baseline circulatory NLR is a strong predictor of survival outcomes after radio(chemo)therapy for patients with HNSCC [38–42]. Cancer is a systemic disease whose course and prognosis is influenced by inflammatory reactions in the micromillieu, as a hallmark of cancer by tumor invasion and metastasis [12]. Two decades ago, Balkwill et al. reported that inflammatory cells and cytokines in the tumor could contribute to tumor growth, progression, and immunosuppression [43]. Since then, several HPs, such as platelets, neutrophils, lymphocytes, NLR, PLR, and MPV, have been studied in various malignant tumors for their effects on tumor pathology [44–48]. In patients with HNSCC, numerous analyses have been performed concerning PET/CT-MPs regarding tumor control and prognosis, reporting MTV defined from pretreatment FDG-PET scans as the strongest predictor of patient outcome after radio(chemo)therapy [49]. The effect of pretreatment SUVmax and SUVmean on patient outcome seems to be a less important factor compared with MTV [49].

There is no clear answer on the relationship between systemic inflammatory parameters and outcome. A possible explanation is that distant (micro)metastases are already manifest at initial diagnosis, which cannot yet be detected by any imaging modality [50]. For clinical routine, it might be interesting to compute a score comprising PET/CT-MPs and HPs for the prediction of already existing but subclinical distant metastases, in order to adapt individual therapy concepts.

In addition to its retrospective design, this hypothesis-generating study has a number of limitations. Using our small cohort, we did not observe a correlation between NLR and PET/CT-MP. Studies with larger or pre-defined cohort sizes are required to further test these potential relationships, because of a substantial type II error risk due to our under-powered sample size. At the time patients in this study were examined, HPV status was not routinely acquired, and we are unable to assess the influence of HPV status on the parameters measured in this study. Furthermore, the effect of some PET/CT-MPs, in particular MTV, could be influenced by the tumor lesion's size, similar to the influence of tumor volume on the TNM stage [51, 52]. For example, the relationship with PLR could be a result of the platelets' contribution to tumor growth [53]. Moreover, we have only recorded PET/CT-MPs for the primary lesions, and other FDG-avid metastatic lesions should be considered by future studies, ideally of prospective design. Finally, although our historical patient collective (2007–2010) afforded a follow-up for clinical outcomes, the scanner type used does not represent the state of the art, where digital and long axial field-of-view PET/CT systems can show improved lesion quantification [54].

## Conclusions

In patients with HNSCC treated with definitive radio(chemo)therapy or oncologic surgery followed by adjuvant RT, baseline PLR is correlated with MTV and TLG but not with SUV. NLR did not correlate with any PET/CT-MPs analyzed in our study. Confirmatory studies and further investigation concerning immunohistochemistry in HNSCC are needed, and a potential interaction between tumor microenvironment, host inflammation, and FDG-PET/CT measures warrants more detailed research, maybe yielding information on tumor behavior and decision-making in radioimmunotherapeutic or treatment deescalating options.

## Supplementary Information

**Additional file 1. Fig. S1**Scatter diagram for correlation between PET and hematological parameters excluding oral cavity cancers from the analysis. Abbreviations: SUVmax = maximum standardized uptake value; NLR = neutrophil-to-lymphocyte ratio.

**Additional file 2. Fig. S2**Scatter diagram for correlation between PET and hematological parameters in oral cavity cancers only. Abbreviations: SUVmax = maximum standardized uptake value; NLR = neutrophil-to-lymphocyte ratio.

## Data Availability

The datasets used or analyzed during the current study are available from the corresponding author on reasonable request.

## Abbreviations

N: Patient number
PET/CT: Positron emission tomography/computed tomography
MP: Metabolic parameter
HP: Hematological parameter
NLR: Neutrophil-to-lymphocyte ratio
PLR: Platelet-to-lymphocyte ratio
SUVmax: Standardized uptake value
MTV: Metabolic tumor volume
TLG: Tumor lesion glycolysis
LMR: Lymphocyte-to-monocyte ratio
MLR: Monocyte-lymphocyte ratio
ICH: Immunohistochemistry
ALN: Axillary lymph node
PFS: Progression-free survival
OS: Overall survival
RFS: Recurrence-free survival
SCC-ag: Squamous cell carcinoma antigen
mGPS: Modified glasgow prognostic score
EGFR: Epidermal growth factor receptor
TAMs: Tumor-associated macrophages
MPV: Mean platelet volume
Na: Not applicable
UICC: Union for International Cancer Control
RT: Radiotherapy
HNSCC: Head and neck squamous cell carcinoma
TILs: Tumor-infiltrating lymphocytes
NSCLC: Non-small-cell lung carcinoma
FDG 18: F fluorodeoxyglucose
PET/CT: Positron-emission tomography/computed tomography
MPs: Metabolic parameters
PD-L1: Programmed cell death ligand-1
