# Supplementary figures and images for: Correlation between hematological parameters and PET/CT metabolic parameters in patients with head and neck cancer
Source: Radiat Oncol. 2022 Aug 13;17:141. doi: 10.1186/s13014-022-02112-4 (PMC9375277; doi:10.1186/s13014-022-02112-4)

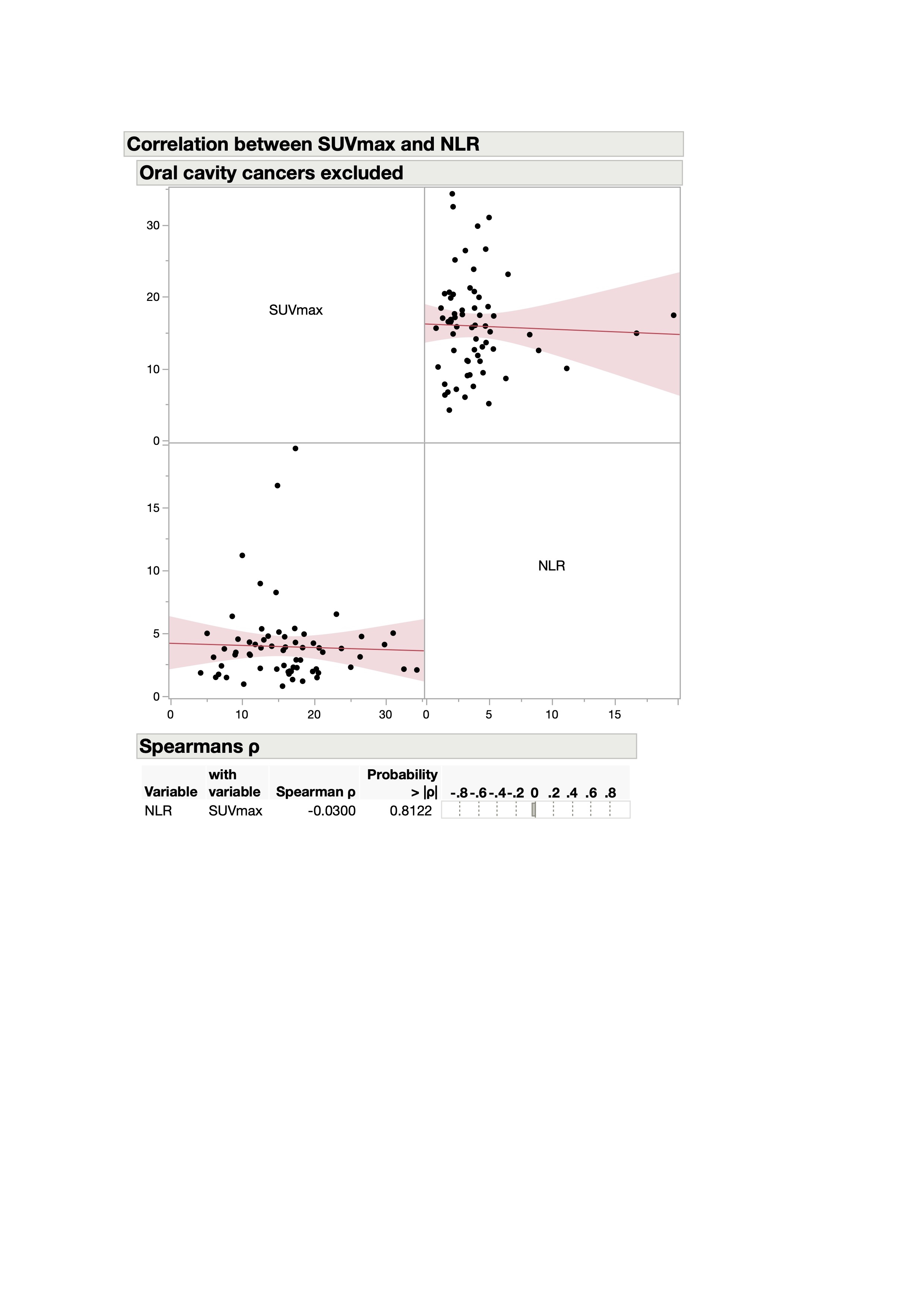

Supplement: Supplementary file 1 — Additional file 1. Fig. S1Scatter diagram for correlation between PET and hematological parameters excluding oral cavity cancers from the analysis. Abbreviations: SUVmax = maximum standardized uptake value; NLR = neutrophil-to-lymphocyte ratio. [file 13014_2022_2112_MOESM1_ESM.jpg]

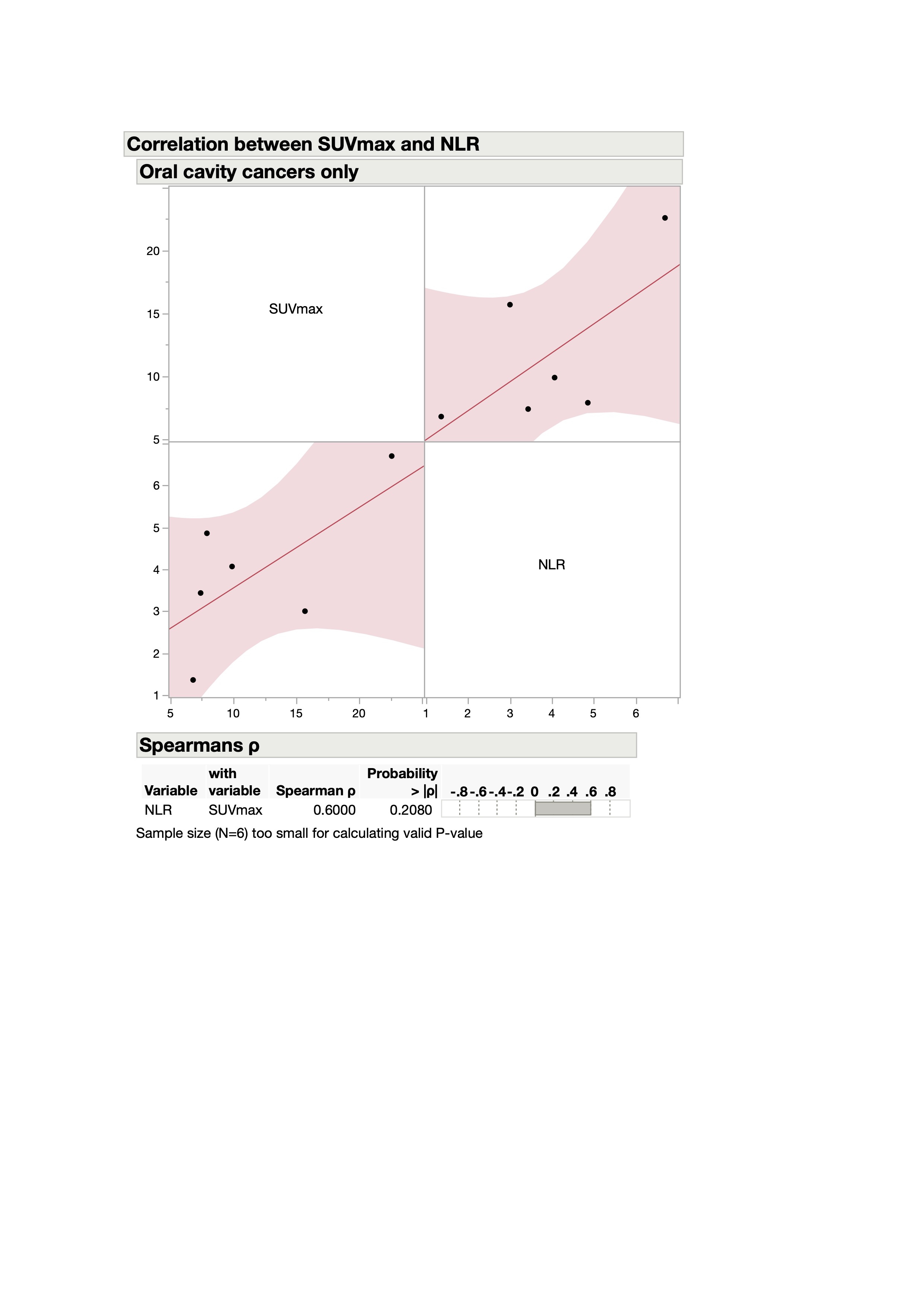

Supplement: Supplementary file 2 — Additional file 2. Fig. S2Scatter diagram for correlation between PET and hematological parameters in oral cavity cancers only. Abbreviations: SUVmax = maximum standardized uptake value; NLR = neutrophil-to-lymphocyte ratio. [file 13014_2022_2112_MOESM2_ESM.jpg]
